# Imaging spectrum of spinal dysraphism: A diagnostic challenge

**DOI:** 10.4102/sajr.v27i1.2747

**Published:** 2023-11-27

**Authors:** Mohit K. Shrivastva, Mousam Panigrahi

**Affiliations:** 1 Department of Radiodiagnosis, Rohilkhand Medical College and Hospital, Bareilly, Uttar Pradesh, India

**Keywords:** spinal dysraphism, myelomeningocoele, lipomyelomeningocoele, lipomyelocoele, diastematomyelia, hemimyelomeningocoele, caudal regression syndrome, dorsal dermal sinus

## Abstract

Spinal dysraphism (SD) is a collective term for congenital malformations of the spine and spinal cord. It includes a wide range of congenital anomalies resulting from aberrations in the stages of gastrulation, primary neurulation and secondary neurulation. Spinal dysraphism may lead to neurological impairment of varying severity including weakness of the extremities, incontinence of bowel and bladder, sexual dysfunction, among others. Diagnosis of SDs is quite challenging because of its wide spectrum and complex cascade of embryologic events. Knowledge of normal embryology and proper understanding of imaging features of SD are important for early accurate diagnosis.

**Contribution:**

This series of five cases describes the imaging spectrum of spinal dysraphism and highlights the embryological basis for their development, which could facilitate early correct diagnosis, surgical planning and reduced morbidity related to these malformations. It also includes an extremely rare case of complex spinal dysraphism (Type II diastematomyelia with right hemimyelomeningocoele and left hemilipomyelomeningocoele) with Chiari II malformation.

## Introduction

Spinal dysraphism (SD) is a collective term for congenital malformations of the spine and spinal cord.^1^ It includes a wide range of congenital anomalies resulting from aberrations in the stages of gastrulation, primary neurulation and secondary neurulation.^2^ Spinal dysraphisms have a prevalence of ~1 to 3 per 1000 live births with the lumbosacral spine being the most common site.^1^ Spinal dysraphisms may lead to neurological impairment of varying severity including weakness of the extremities, incontinence of bowel and bladder, and sexual dysfunction, among others.^1^

The diagnosis of SDs is quite challenging because of its wide spectrum and complex cascade of embryologic events. However, early detection of SD is possible with proper understanding of the imaging features of the SD spectrum, which allows parent counselling, appropriate treatment planning and reduced morbidity related to these malformations. MRI is the modality of choice for diagnosis, surgical planning and postoperative assessment of SDs because of its high spatial resolution and tissue contrast.^1^

## Case series

### Case 1

**FIGURE 1:**
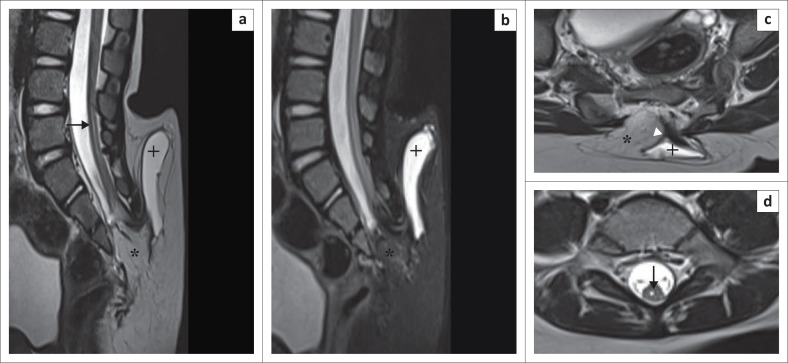
Lipomyelomeningocoele. Sagittal T2-weighted image (T2WI) (a) and sagittal short tau inversion recovery (STIR) (b) demonstrate a low-lying spinal cord tethered to a lipoma (*). Axial T2WI (c) shows protrusion of neural tissue (white arrowhead) and an enlarged cerebrospinal fluid (CSF) space (**+**) through the bony defect in the posterior elements. The cord–lipoma interface is present outside the spinal canal. The overlying skin is intact. Syrinx formation (black solid arrow) is noted (a, d) at the L5 and S1 vertebral levels.

A 2-year-old girl presented with lower back swelling since birth. The lumbosacral spine MRI revealed a low-lying spinal cord (tip at the S3 vertebral level). Syrinx formation was seen in the spinal cord at the L5 and S1 levels. A bony defect was seen in the posterior elements at the S3 level. There was protrusion of neural elements and an enlarged cerebrospinal fluid (CSF) space projecting posteriorly through the defect into the subcutaneous plane. A lipomatous mass was seen extending from the subcutaneous tissue into the spinal canal via the bony defect. The cord–lipoma interface was outside the vertebral canal. The overlying skin and subcutaneous tissue were intact (Figure 1). A diagnosis of lipomyelomeningocoele was made. The patient was referred to a neurosurgeon for surgical management.

### Case 2

**FIGURE 2:**
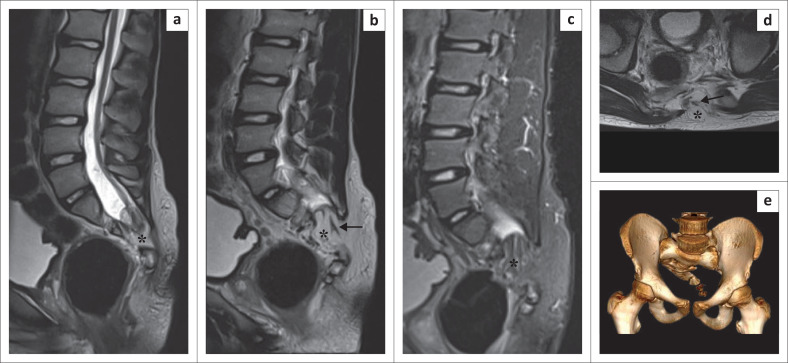
Lipomyelocoele. Sagittal T2-weighted image (T2WI) (a) shows a low-lying spinal cord tethered to a lipoma (*). The lipoma is extending into the spinal canal and is continuous with the subcutaneous fat posteriorly on sagittal T2WI (b), sagittal short tau inversion recovery (STIR) (c) and axial T2WI (d). Sagittal and axial T2WI (b, d) show the cord–lipoma interface (black solid arrow) at the level of the neural arches, without widening of the subarachnoid space. Volume rendered CT image (e) demonstrates a deformed sacrum.

A 13-year-old male presented with pain and stiffness in the lower back with bilateral lower limb weakness for 4 years. MRI lumbosacral spine revealed hypoplasia of the left half of sacrum. Bony defects were noted in the posterior elements of the S1, S2 and S3 vertebrae. A lumbosacral transitional vertebra, Castellvi type IIa, was seen. Bony abnormalities were confirmed with a CT scan. The spinal cord was low lying (lower end at S1–S2 level) and tethered to a subcutaneous lipoma that was penetrating the spinal canal through the defects in the S2 and S3 vertebrae. The cord–lipoma interface was at the level of the neural arch. The overlying skin was intact (Figure 2). A diagnosis of lipomyelocoele with a deformed sacrum was made. Surgical management (rectification of tethered cord and resection of the lipomatous component) was done. The patient’s symptoms improved following surgery.

### Case 3

**FIGURE 3:**
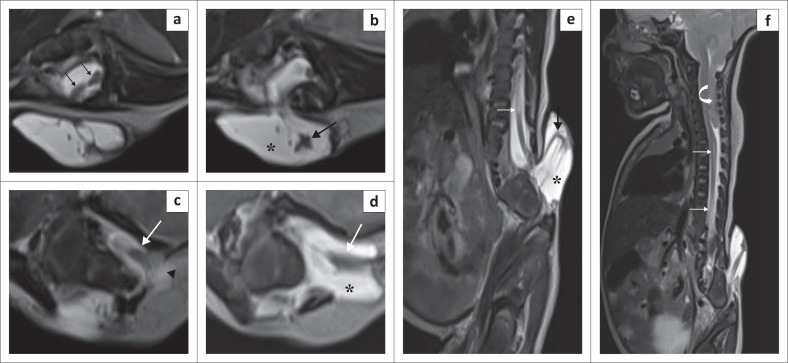
Type II diastematomyelia with a right hemimyelomeningocoele, left hemilipomyelomeningocoele and Arnold Chiari II malformation. Axial T2-weighted image (T2WI) (a) shows the splitting of spinal cord (diastematomyelia) (thin black arrows). Axial and sagittal T2WI (b & e) show herniation of the right hemicord (solid black arrows) along with an enlarged subarachnoid space (*) through the right posterior bony defect with no overlying skin or subcutaneous tissue (right hemimyelomeningocoele). Axial T2WI (c) shows herniation of the left hemicord (solid white arrow) through the left posterior bony defect with tethering to a subcutaneous lipomatous mass (black arrowhead). Axial T2WI (d) shows protrusion of the neural tissue and an enlarged CSF space (*) through the bony defect (left hemilipomyelomeningocoele). Sagittal T2WI (f) shows herniation of the cerebellum (white curved arrow) into the spinal canal through the foramen magnum and syrinx formation (thin white arrow).

A 9-day-old boy presented with scoliosis and swelling over his lower back since birth. MRI spine revealed bony defects in posterior elements of multiple vertebrae in the lumbosacral region. The spinal cord was low lying with splitting of the spinal cord in the lower lumbar region (Type II diastematomyelia). The right hemicord was seen herniating along with an enlarged CSF space through the defect in the posterior elements at the S1 level. The neural placode was elevated above the level of the skin by the enlarged CSF space with discontinuity of the skin and subcutaneous tissue over the herniated tissue. The left hemicord was herniating along with an enlarged CSF space through the defect in the posterior elements at the S1 level and tethered to subcutaneous lipomatous tissue. The cord–lipoma interface was outside the spinal canal. The overlying skin and subcutaneous tissue were intact on the left side. There was herniation of the cerebellum across the foramen magnum into the spinal canal up to the C7 level. Cord syrinx formation was seen in the thoracic and lumbar levels (Figure 3). A diagnosis of complex spinal dysraphism (Type II diastematomyelia with right hemimyelomeningocoele and left hemilipomyelomeningocoele) with Arnold Chiari II malformation was made. It was an extremely rare case of complex spinal dysraphism with hemimyelomeningocoele and hemilipomyelomeningocoele in the same patient.^1,2,3,4,5^ To the best of our knowledge, no such case has been published in the literature to date. The patient was referred to a higher centre for further management.

### Case 4

**FIGURE 4:**
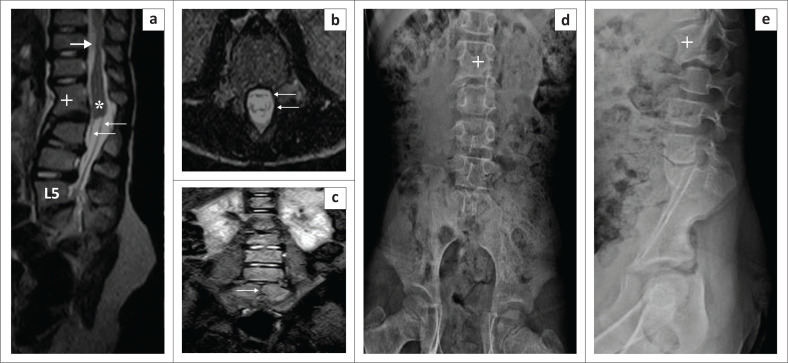
Type I caudal regression syndrome. Sagittal T2-weighted image (T2WI) (a) and radiographs (d, e) show non-visualisation of the sacrum (beyond S1) and coccyx with a L1–L2 block vertebra (**+**). There is mild anterior wedging of the L3 vertebra. Coronal short tau inversion recovery (STIR) (c) shows a vertical midline cleft (thin black arrow) in the S1 vertebral body. Sagittal T2WI (a) shows attenuation of the spinal cord (solid white arrow) from the T10 to T12 vertebral levels and a thickened conus medullaris (*) with abrupt termination at the lower end of the block L1–L2 vertebra. The nerve roots follow an atypical course having a ‘double-bundle shape’ (a, b) (thin white arrows).

A 6-year-old girl presented with weakness of the lower limbs and difficulty in walking since childhood. Plain radiographs of the lumbosacral spine revealed non-visualisation of the sacrum (beyond S1) and coccyx with a L1–L2 block vertebra. There was mild anterior wedging at the L3 vertebral level. MRI lumbosacral spine revealed a vertical midline cleft in the S1 vertebral body. There was thinning of the spinal cord from the T10 to the T12 vertebral level. The conus medullaris was thickened with abrupt termination at the lower end of the L1–L2 block vertebra. The nerve roots followed an atypical course having a ‘double-bundle shape’ (Figure 4). A diagnosis of type I caudal regression syndrome (CRS) was made. The patient was treated conservatively with regular follow-up.

### Case 5

**FIGURE 5:**
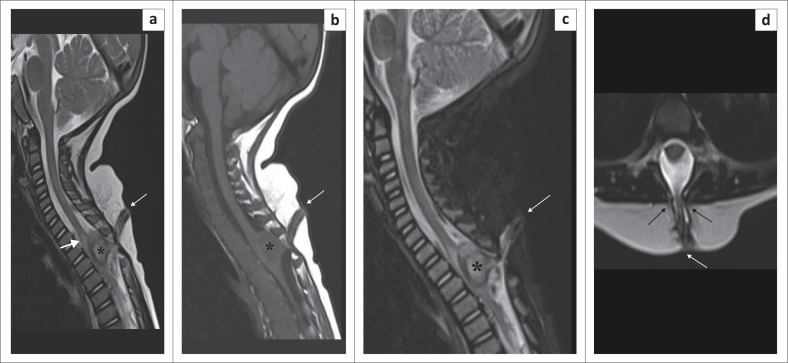
Dorsal dermal sinus. Sagittal T2-weighted image (T2WI) (a), T1WI (b), short tau inversion recovery (STIR) (c) and axial T2WI (d) show a sinus track (thin white arrow) extending from the skin surface to the thoracic spinal cord through a posterior bony defect (thin black arrows in d) at the T3 vertebral level. A heterogeneous lobulated intradural extramedullary collection (*) is causing significant spinal cord compression with associated spinal cord oedema/myelomalacia (solid white arrow).

An 18-month-old boy presented with fever, pain and a discharging sinus in the midline in the upper back associated with bilateral lower limb weakness for 15 days. An ostium was present in the upper back region since birth. MRI cervicothoracic spine revealed non-visualisation of the spinous process of the T3 vertebra. A sinus track was seen extending from the skin surface to the spinal canal through this defect and ending in a heterogeneous lobulated intradural extramedullary lesion appearing mildly hyperintense on T2 short tau inversion recovery (STIR) and hypointense on T1-weighted image (T1WI). The lesion was causing significant cord compression with associated cord oedema/myelomalacia (Figure 5). A diagnosis of the dorsal dermal sinus (DDS) with secondary abscess formation was made. Surgical resection of the tract and abscess drainage was performed.

## Discussion

Spinal dysraphism includes various abnormalities in the development of spinal cord during the 2nd to 6th weeks of gestation relating to partial midline closure of osseous, nervous and mesenchymal tissues.^1^

### Normal embryology of spinal cord (Figure 6)

Gastrulation, primary neurulation and secondary neurulation are the three fundamental stages that constitute the development of the spine and spinal cord.^1^

**FIGURE 6:**
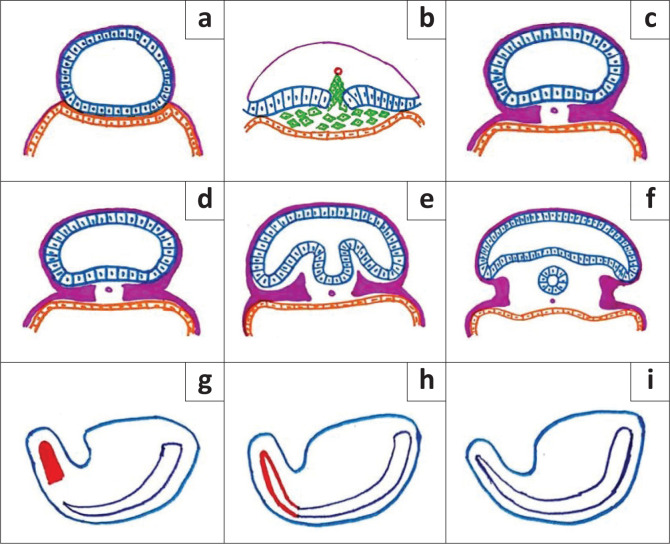
*Gastrulation* (a–c): (a) The hypoblast (orange cells) and the epiblast (blue cells) form the bilaminar embryonic disc. (b) Hypoblast forms the endoderm after being displaced caudally by cells (green cells) arising from the primitive pit (red circle) entering between the epiblast and hypoblast. (c) Mesoderm (magenta) and notochord (magenta circle) are formed by cells migrating bilaterally above the endoderm. Trilaminar disc and notochord are formed. *Primary neurulation* (d–f): (d) Formation of the neural plate is stimulated by the notochord in the dorsal midline of the ectoderm. (e) Two neural folds with neural groove in between are formed. (f) The neural folds fuse in the midline and detach from the cutaneous ectoderm, forming the neural tube. *Secondary neurulation* (g–i): (g) Caudal to the primary neural tube, tail bud (red) is formed by a solid mass of totipotent cells. (h) Secondary neural tube is formed by internal cavitation in the tail bud. (i) Secondary neural tube joins the primary neural tube (deep blue) creating a continuous structure.

*Gastrulation* is the process of addition of mesoderm between the epiblast and hypoblast of the bilaminar embryonic disc leading to the formation of the trilaminar embryonic disc during the second and third gestational weeks. The notochord is also formed at this stage.^1^

*Primary neurulation*: During 3rd and 4th week of gestation, the formation of the neural plate is stimulated by the notochord in the dorsal midline of the ectoderm which is followed by the formation of two neural folds with the neural groove in between.^1^ The neural folds fuse forming a cylindrical neural tube. Closure of the neural groove proceeds bidirectionally in a zipper-like manner with the cranial and the caudal ends closing on days 25 and 27/28, respectively.^6^

*Secondary neurulation* occurs during the 5th and 6th week of gestation.^1^ Caudal to the primary neural tube, the tail bud is formed by a solid mass of totipotent cells. The secondary neural tube is formed by internal cavitation in the tail bud. This secondary neural tube joins the primary neural tube creating a continuous structure.^7^ By retrogressive differentiation, the tail bud regresses and forms the filum terminale (FT) and the tip of the conus medullaris.^7^ Deviation in any of these steps can lead to SD.

### Clinical-radiologic classification of spinal dysraphisms (Figure 7)^1^

#### Open spinal dysraphisms

**Myelomeningocoele:** A myelomeningocoele is characterised by exposure of the neural placode to the exterior with widening of the underlying subarachnoid space that protrudes through the neural arch defect raising the placode above the skin surface in the midline of the back. It is the most common type of open SD with a prevalence of ~ 0.6–1.0 per 1000 live births.^1^ The lumbosacral region is most commonly affected. The embryologic basis of a myelomeningocoele is explained by a primary failure in closure of the neural tube, a defect in primary neurulation. A part of the neural plate persists in its primitive stage, failing to form the neural folds and neural groove, resulting in the placode. Margins of the adjacent cutaneous ectoderm stay tethered to the non-neurulated neural plate segment that fails to fuse and form the future skin. Mesenchymal migration behind the neural tube is restricted because of the non-separation of the neuroectoderm from the cutaneous ectoderm, causing a defect in the neural arch.^1^

**FIGURE 7:**
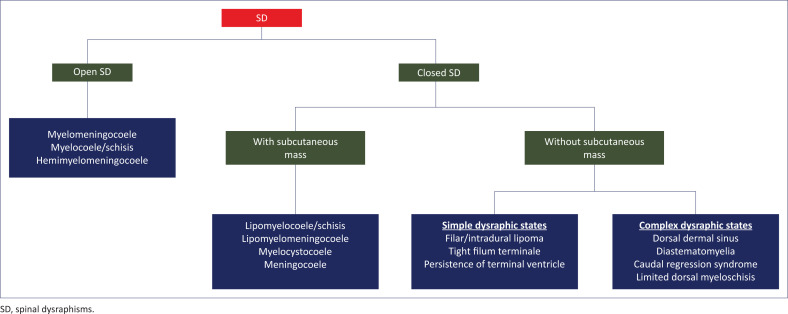
Clinical-radiologic classification of spinal dysraphisms.

**Myelocoele and myeloschisis:** In a myelocoele, the placode is subjected to the outside through a spina bifida without enlargement of the subarachnoid space.^1^ Its development is similar to that of myelomeningocoele.^7^ As there is no enlargement of the subarachnoid space beneath, the placode is flush with the skin surface (myelocoele) or depressed (myeloschisis).^1^

**Hemimyelomeningocoele or hemimyelocoele:** Hemimyelo-meningocoele is an extremely rare condition resulting from defective gastrulation with failed midline notochordal integration and an additional fault in the primary neurulation of one hemicord. In hemimyelo-meningo-coele, there is a widening of the subarachnoid space posteriorly raising the hemiplacode above the skin surface, whereas in hemimyelocoele, the hemiplacode is flush with the skin surface.^1,3,4^

#### Closed spinal dysraphisms with a subcutaneous mass

**Lipomyelomeningocoele:** A subcutaneous lipoma coupled with a posterior myelomeningocoele constitutes a lipomyelomeningocoele. Early disjunction of the cutaneous ectoderm and the neuroectoderm during primary neurulation results in lipomyelomeningocoele. As a result, mesenchymal tissue can migrate into the neural tube and form a lipomatous mass that prevents effective neurulation. MRI shows expansion of the subarachnoid space with neural elements entering the meningocoele and attached to a subcutaneous lipoma. The cord–lipoma junction is outside the spinal canal.^1^

**Lipomyelocoele and lipomyeloschisis:** A subcutaneous lipoma enters the spinal canal through a defect in the posterior neural arch and anchors to the tethered cord. Development of these is similar to that of lipomyelomeningocoele. There is no subarachnoid space enlargement or meningeal herniation. The position of the cord–lipoma interface differentiates these two conditions. The cord–lipoma junction could be within the spinal canal (lipomyeloschisis) or at the level of the neural arches (lipomyelocoele).^1^

**Meningocoele:** Meningocoele is the herniation of an enlarged subarachnoid space lined by meninges through a spina bifida without any neural component. The meninx primitiva and cutaneous ectoderm fail to separate and protrude via the neural arch defect, encouraged by CSF pulsation.^1^

**Myelocystocoele:** A hydrosyringomyelic cavity herniating through the neural arch defect and into a meningocoele is the hallmark of myelocystocoele. There are two types: terminal (lumbosacral region – more common) and non-terminal (cervical or thoracic segment).^1,8^ In terminal myelocystocoele, the surrounding mesenchymal tissue ruptures because of the expansion of the cavity of the persistent secondary neural tube. The meningocoele is a continuation of the subarachnoid space, while the hydrosyringomyelic cavity is continuous with the ependymal canal. Partial closure of the neural tube and non-separation of the ectoderm from the neuroectoderm lead to non-terminal myelocystocoele. A fibroneurovascular filament develops from the spinal cord’s posterior wall, passes through the meningocoele and the dural opening and then attaches to aberrant skin. A hydrosyringomyelic cavity is formed by the expansion of the ependymal canal’s posterior wall through the neural arch defect and meningocoele, encouraged by continuous CSF pulsation.^1^ In non-terminal myelocystocoele, the spinal cord’s anterior wall remains contained in the vertebral canal, while the hydrosyringomyelic cavity’s posterior wall herniates through the spina bifida into the meningocoele.^7,9^

#### Closed spinal dysraphisms without a subcutaneous mass

##### Simple dysraphic states

***Intradural lipoma*:** Adipose cells inside the dural sac constitute this benign lesion.^10,11^ The embryologic mechanism is similar to that of the lipomyelocoele. A subpial lipomatous lesion is seen on MRI lying between the fibres of the placode.^1^

***Lipoma of the filum terminale*:** Improper cavitation of the tail bud with continued presence of cells that can mature into adipocytes after separation of the neuroectoderm from the cutaneous ectoderm leads to a filum terminale (FT) lipoma.^5^ Filar lipoma is seen on MRI as a small T1 and T2 hyperintense lesion in the region of FT without any communication with the medullary cone.^1^

***Persistence of the terminal ventricle (fifth ventricle)*:** This is a small ependymal-lined cavity centrally located within the conus medullaris.^12^ The fifth ventricle is formed during secondary neurulation because of partial regression of the terminal ventricle. It remains continuous with the central canal of the spinal cord.^5^ It follows CSF signal intensity on all MRI sequences without any post-contrast enhancement.^1^

##### Complex dysraphic states

***Dorsal dermal sinus:*** A dorsal dermal sinus (DDS) is a midline epithelium-lined fistula connecting the central nervous system or its meninges with the skin surface. The most common site of involvement is the lumbosacral region.^8^ A dorsal dermal sinus occurs during primary neurulation because of non-separation of cutaneous ectoderm and neuroectoderm. An ostium is seen in the midline of the skin surface on clinical inspection. MRI reveals an oblique tract from the skin surface to the spinal canal traversing through the subcutaneous tissue. It may be associated with an ectodermal inclusion cyst, dermoid cyst or abscess.^1^

***Diastematomyelia:*** Diastematomyelia is the splitting of the spinal cord into two hemicords, each with its central canal and pial covering. During gastrulation, failure of midline integration of the two paired notochordal anlagen leads to two hemicords over a variable segment with the intervening space occupied by totipotent primitive streak cells.^13^ In Type I, a bony or cartilaginous septum is developed from the intervening primitive streak forming two dural sacs. In Type II, a single dural sac is seen encasing both hemicords because of regression of the primitive streak or formation of a fibrous septum.^1^

***Caudal regression syndrome:*** Caudal regression syndrome (CRS) is characterised by partial or complete agenesis of the spinal column associated with pulmonary hypoplasia, imperforate anus, genito-urinary anomalies and limb abnormalities. The incidence of CRS is 1.3 per 10 000 newborns.^1^ Caudal regression syndrome results from abnormal tail bud development that interferes with normal neuronal and mesodermal cell migration, causing irregularities in skeletal development and associated anomalies.^14^ Caudal regression syndrome is of two types. In type I, there is low thoracic to coccygeal region vertebral dysgenesis with an abrupt termination of the conus medullaris associated with parallelism (‘double-bundle shape’) of the cauda equina nerve roots. Type II CRS demonstrates segmental agenesis of the sacrum or the coccyx with the spinal cord invariably attached to an intraspinal mass.^1^

## Conclusion

Spinal dysraphism is a heterogeneous and complex group of abnormalities occurring because of derangements in spinal development. Because of cognitive impairment and accompanying neural abnormalities, SD is a significant contributor to childhood morbidity, with major economic and psychosocial ramifications. The diagnosis and presurgical assessment of SDs depend heavily on neuroimaging. However, the imaging features of SD may appear complicated. Therefore, a rational approach based on the correlation of clinical, embryological and neuroimaging information considerably aids the diagnosis in the vast majority of cases.
